# The use of a dedicated neurological triage system improves process times and resource utilization: a prospective observational study from an interdisciplinary emergency department

**DOI:** 10.1186/s42466-019-0036-y

**Published:** 2019-10-25

**Authors:** Carolin Hoyer, Patrick Stein, Hans-Werner Rausch, Angelika Alonso, Simon Nagel, Michael Platten, Kristina Szabo

**Affiliations:** 1 Department of Neurology, UniversitätsMedizin Mannheim, Heidelberg University, Medical Faculty, Theodor-Kutzer-Ufer 1-3, 68135 Mannheim, Germany; 2 Department of Neurology, University Hospital, Heidelberg University, Heidelberg, Germany

**Keywords:** Emergency department, Neurology, Triage

## Abstract

**Background:**

Patients with neurological symptoms have been contributing to the increasing rates of emergency department (ED) utilization in recent years. Existing triage systems represent neurological symptoms rather crudely, neglecting subtler but relevant aspects like temporal evolution or associated symptoms. A designated neurological triage system could positively impact patient safety by identifying patients with urgent need for medical attention and prevent inadequate utilization of ED and hospital resources.

**Methods:**

We compared basic demographic information, chief complaint/presenting symptom, door-to-doctor time and length of stay (LOS) as well as utilization of ED resources of patients presenting with neurological symptoms or complaints during a one-month period before as well as after the introduction of the Heidelberg Neurological Triage System (HEINTS) in our interdisciplinary ED. In a second step, we compared diagnostic and treatment processes for both time periods according to assigned acuity.

**Results:**

During the two assessment periods, 299 and 300 patients were evaluated by a neurologist, respectively. While demographic features were similar for both groups, overall LOS (*p* < 0.001) was significantly shorter, while CT (*p* = 0.023), laboratory examinations (*p* = 0.006), ECG (*p* = 0.011) and consultations (*p* = 0.004) were performed significantly less often when assessing with HEINTS. When considering acuity, an epileptic seizure was less frequently evaluated as acute with HEINTS than in the pre-HEINTS phase (*p* = 0.002), while vertigo patients were significantly more often rated as acute with HEINTS (*p* < 0.001). In all cases rated as acute, door-to-doctor-time (DDT) decreased from 41.0 min to 17.7 min (*p* < 0.001), and treatment duration decreased from 304.3 min to 149.4 min (*p* < 0.001) after introduction of HEINTS triage.

**Conclusion:**

A dedicated triage system for patients with neurological complaints reduces DDT, LOS and ED resource utilization, thereby improving ED diagnostic and treatment processes.

## Introduction

Emergency department (ED) utilization in many countries has substantially increased in the last two decades [32]. In Germany, the number of referrals has risen to approximately 20 million over the past several years and is expected to increase further [43]. Among emergency presentations, the number of patients with neurological disturbances and disorders has grown considerably, currently accounting for approximately 15% of ED admissions [39, 41]. The emergency care of these patients poses particular challenges at the specific interface between pre-hospital evaluation and acute care in the ED. First, assessment of these patients can be difficult for prehospital first-aid and non-neurological emergency care providers due to the wide variety of non-specific symptoms of potentially broad etiology and thus varying degrees of clinical significance [24, 25]. This may lead emergency medical services to err on the side of safety and apply a low threshold for transporting patients with neurological symptoms to the ED [6] when this would not have been necessary. Consequently, neurological ED patients are less frequently admitted to hospital when compared to general medicine, vascular and neurosurgery patients [34, 38]. A further illustration of this “safety thinking” in the context of neurological symptoms is provided by study a by Robertson et al., who found that only one third of patients referred for neurological assessment to a rapid referral neurological acute clinic were retrospectively considered to have warranted an urgent evaluation [35]. It follows that gate-keeping practices need to be established in order to adequately and economically allocate limited ED resources. At the same time, however, treatment of patients presenting with neurological emergencies such as an acute ischemic stroke, cerebral hemorrhage, status epilepticus or meningoencephalitis is often time-sensitive and requires immediate recognition and swift management in the ED [4, 9, 30, 40]. This two-faceted scenario is ideal for the application of a triage procedure since triage systems were developed to facilitate acuity assessment as well as to predict patient disposition and resource utilization [11, 22]. However, neurological symptoms appear to be inadequately represented in established triage systems like the Manchester Triage System (MTS) or the Emergency Severity Index (ESI): While there are no dedicated investigations into the performance of currently used systems in neurological patients, Lange et al. found that over 50% of ED neurological patients were triaged into ESI category 2, which allows for a door-to-doctor time of up to 10 min [16]. Granting this amount of latency in door-to-doctor time most likely impacts on the quality of acute care in many patients with neurological emergencies, most notably in acute ischemic stroke, where a door-to-needle time of 30 min or less [12, 18] may be difficult to achieve under these circumstances. At the same time, categorizing a substantial number of patients with different degrees of symptom acuity and relevance as second most-urgent according to the ESI generates the need for a “triage within triage” in times of high ED patient traffic, thus leading the concept to some degree *ad absurdum*. Finally, the past several years have seen a number of developments in acute stroke care [3, 28], which, given the extended time-window for intervention under certain circumstances, further necessitates the need for a distinguished approach to the patient with an acute neurological deficit. A dedicated neurological triage method, the Heidelberg Neurological Triage System (HEINTS), was developed in order to address these issues [31]. This tool reliably detected neurological emergencies in a validation study but, hitherto, has not yet been applied in an interdisciplinary emergency room setting. We hypothesize that the use of HEINTS as a specific neurological triage tool influences process times and resource utilization in the emergency assessment of patients presenting with neurological complaints or symptoms in an interdisciplinary emergency department.

## Methods

### Study design

We analyzed records of patients who consecutively presented or were referred to the Interdisciplinary Emergency Department (IED) of the University Medical Centre, Mannheim, Germany, between April and Mai 2017, and between April and May 2018 for neurological consultation. In the Mannheim IED, at least one neurology resident is present 24/7 either for first-line assessment if prehospital evaluation suggests a neurological condition, or second-line as per judgement of a non-neurologist IED physician.

**Fig. 1:**
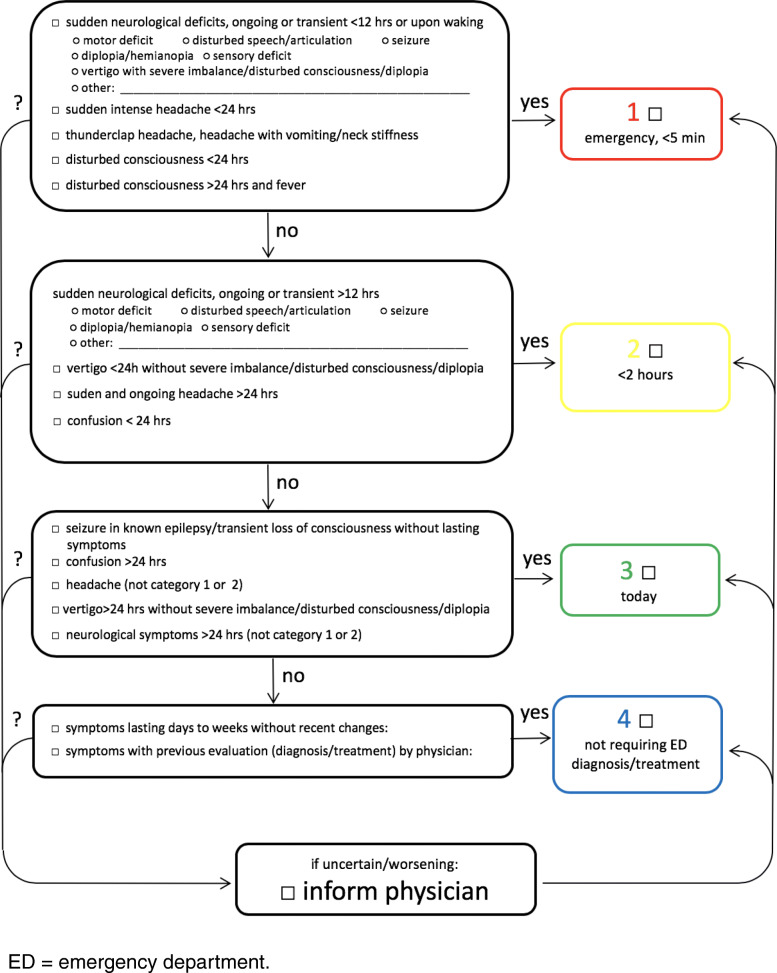
The Heidelberg Neurological Triage System (HEINTS). ED = emergency department

In February 2018, HEINTS, a dedicated neurological triage tool, was established in the IED, and every patient presenting with a neurological symptom as chief complaint was triaged accordingly (Fig. 1). Prior to the introduction of HEINTS, the ED neurologist was informed about every new patient with neurological symptoms immediately upon arrival, and target times for door-to-doctor contact were specified by internal guidelines, which in turn were established with close reference to official recommendations and guidelines for the emergency management of patients with neurological conditions.

### Analysis of neurological referrals to the IED and assessment of acuity

Data include basic demographic information, chief complaint/presenting symptom [36], door-to-doctor time and length of stay (LOS) as well as utilization of ED resources (computed tomography (CT) including CT angiography (CTA), magnetic resonance imaging (MRI), lumbar puncture (LP), electroencephalography (EEG), laboratory examinations, at least one consultation with another specialty, electrocardiogram (ECG)). The extent of resource utilization for each patient was calculated by adding up diagnostic procedures during ED stay to a sum score (one point each for the ED resources listed above). CT, MRI, LP and EEG were defined as neurology-specific resources.

Two-hundred and ninety-nine patients consecutively presenting to the ED during a 4-week-period in 2017 (“pre-HEINTS” henceforth) were retrospectively evaluated for level of acuity by two experienced neurologists with more than 5 years of practical experience in the IED: Only the history pertaining to the chief complaint and symptoms, any additional pre-existing diagnoses and current medication were presented, on the basis of which it was determined whether the patient was thought to require diagnostic or therapeutic procedures either immediately or within the next 24 h (acute), or whether this was a condition not necessitating immediate or advanced diagnostic procedures in the ED or admission (non-acute; [5]). This decision was made with reference to internal guidelines mentioned previously.

**Fig. 2:**
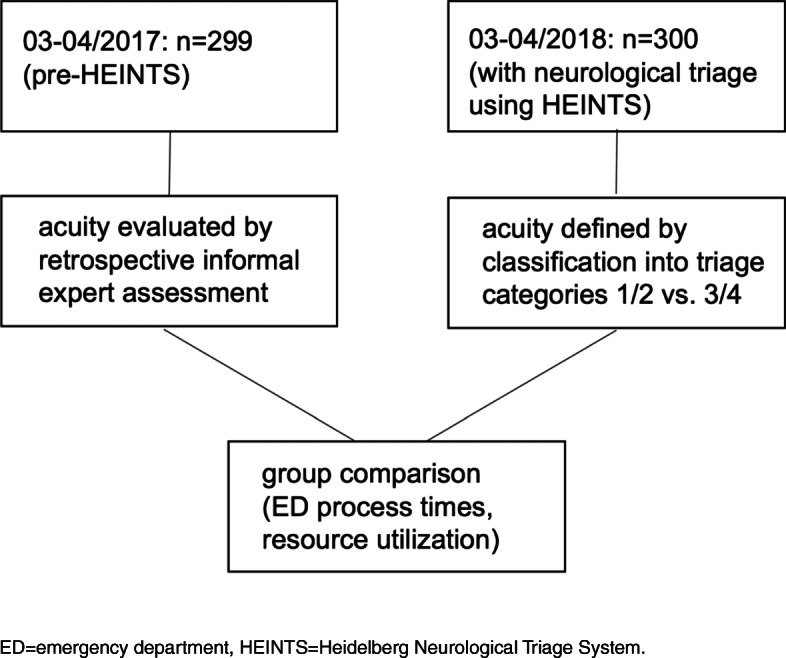
Overview of methods. ED = emergency department, HEINTS=Heidelberg Neurological Triage System

Patients presenting in 2018 (“HEINTS” henceforth) were triaged by the ED neurologist using HEINTS, which categorizes patients with neurological symptoms and complaints into four different categories implying different degrees of urgency for evaluation. Patients triaged to categories 1 and 2 were subsumed under “acute”, whereas patients belonging to categories 3 or category 4 were “non-acute”. Group comparisons were performed between pre-HEINTS and HEINTS patients as well as between subgroups of acute and non-acute patients. For an overview of methods see Fig. 2.

### Statistical analysis

Statistical analysis was performed with STATA® (StataCorp, Texas 77,845, USA, version 11). The distribution of categorial variables between urgent and non-urgent patients was compared by Chi2 tests. Group comparisons of metric data were assessed using independent samples t tests. Somers’ *d* was run to determine the association between door-to-doctor time and ED LOS and HEINTS categories 1–4. A *p*-value of <.05 was considered significant.

## Results

### Characterization of pre-HEINTS and HEINTS patients

No relevant differences were observed with regard to age and gender as well as mode of presentation and disposition (Table 1). Acuity – determined by retrospective specialist evaluation for pre-HEINTS patients and by being assigned to either HEINTS categories 1/2 or 3/4 – did not differ. During the HEINTS period, 94 patients (31.3%) were triaged into the most urgent HEINTS category, 91 patients (30.3%) were triaged to categories 2 and 3 each, and 24 patients (8.0%) were considered to be category 4 patients not in need of ED assessment. Table 2 gives an overview of presenting symptoms in both pre-HEINTS and HEINTS patients with respect to acuity. Significant differences were observed in epileptic seizure with significantly fewer cases evaluated as acute in the HEINTS phase than in the pre-HEINTS phase (4.9% vs. 14.4%; *p* = 0.002) and vertigo where patients were evaluated significantly more often as acute in the HEINTS phase (27.0% vs. 7.2%; *p* < 0.001).

**Table 1:** Characteristics of pre-HEINTS and HEINTS patients

	pre-HEINTS (*N* = 299)	HEINTS (*N* = 300)	*p* value
Demographics			
age, mean (SD)	54,7 (1.24)	56.9 (1.20)	0.186
gender, male, *N* (%)	136 (45.5)	153 (51.0)	0.191
Mode of presentation, *N* (%)			
self-presenting	123 (41.1)	105/298 (35.2)	
EMS ± physician	176 (58.9)	193/298 (64.8)	0.152
Acuity, *N* (%)			
acute	181/295 (61.4)	185 (61.7)	
not acute	114/295 (38.6)	115 (37.3)	1.000
Process times, min (%)			
door-to-doctor time, min (SD)	45.8 (5.91)	36.7 (3.42)	0.186
treatment duration, min (SD)	288.1 (12.57)	136.2 (6.60)	**<.001**
Resource utilization, *N* (%)			
CT	118 (39.5)	92 (30.7)	**0.023**
MRI	67 (22.4)	59 (19.7)	0.409
lumbar puncture	15 (5.0)	9 (3.0)	0.205
laboratory examination	287 (96.0)	273 (91.0)	**0.006**
ECG	188 (62.9)	158 (52.7)	**0.011**
≥ 1 consultation	136 (45.5)	102 (34.0)	**0.004**
Disposition, *N* (%)			
hospital admission	137 (45.8)	139 (46.3)	0.900
discharge	148 (49.5)	149 (49.7)	0.967
DAMA	10 (3.3)	8 (2.7)	0.627
LWBS	4 (1.4)	4 (1.3)	1.000

*CT* computed tomography, *DAMA* discharge against medical advice, *ECG* electrocardiogram, *EMS* emergency medical service, *HEINTS* Heidelberg Neurological Triage System, *LWBS* left without being seen, *MRI* magnetic resonance imaging

**Table 2:** Presenting symptoms of acute and non-acute patients in pre-HEINTS and HEINTS groups

	non-acute			acute		
	pre-HEINTS (*N* = 114)	HEINTS (*N* = 115)	p value	pre-HEINTS (*N* = 181)	HEINTS (*N* = 185)	p value
Presenting symptom, *N* (%)						
ataxia/movement disorder	0	5	0.060	0	5	0.061
disorder of consciousness	3	3	1.000	12	13	1.000
epileptic seizure	22	24	0.767	26	9	**0.002**
headache	22	22	0.974	23	14	0.103
other types of pain	4	0	0.059	1	0	0.498
motor deficit	14	6	0.058	31	37	0.480
confusion/amnesia	1	5	0.213	9	7	0.618
impaired vision	4	3	0.722	9	10	1.000
sensory deficit	9	13	0.381	16	15	0.802
Impaired speech/articulation/						
swallowing	1	5	0.213	26	21	0.436
vertigo	28	19	0.132	13	50	**< 0.001**
other	5	4	0.748	6	3	0.333
non-neurological presenting symptom	4	5	1.000	6	1	0.065
no classification possible	0	1	1.000	0	0	n. a.

*n. a.* not applicable

Regarding overall waiting times, no significant differences emerged when comparing pre-HEINTS and HEINTS patients irrespective of acuity. LOS, however, was significantly shorter in HEINTS patients than in pre-HEINTS patients (pre-HEINTS: 288.1 min ±12.57, triage: 136.2 min ± 6.60; *p* < 0.001). Overall, resource utilization differed between the groups in that fewer resources were used in HEINTS patients (mean 2.34 ± .065 vs. 2.75 ± .063; *p* < 0.001), and mean sum scores of all neurology-specific resources (CT/MRI/EEG/LP) were also lower in HEINTS patients (mean .54 ± .032 vs. .67 ± .035; *p* = 0.005). In particular, CTs, laboratory examinations, ECGs and consultations with other specialties were utilized less frequently during the HEINTS period (Table 1).

### Process times and resource utilization with respect to acuity

**Fig. 3:**
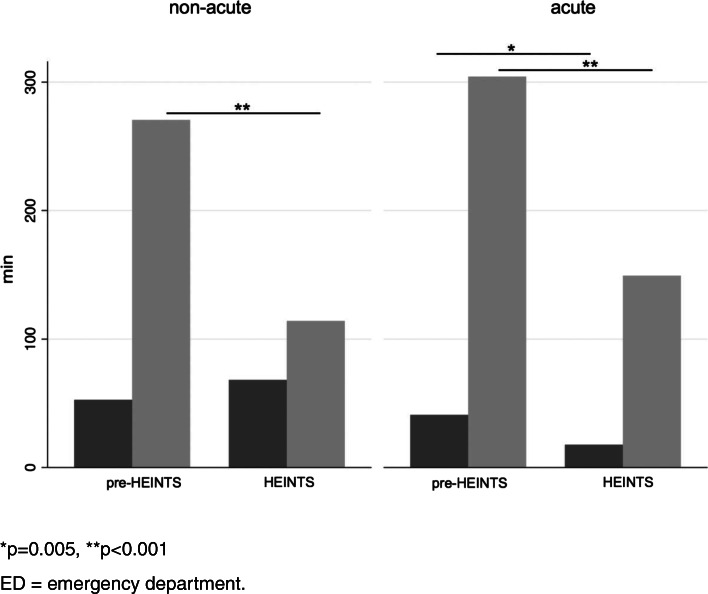
Door-to-doctor time (dark grey) and ED length of stay (light grey) in the emergency department of pre-HEINTS and HEINTS patients with respect to acuity. *p = 0.005, **p < 0.001 ED = emergency department

**Fig. 4:**
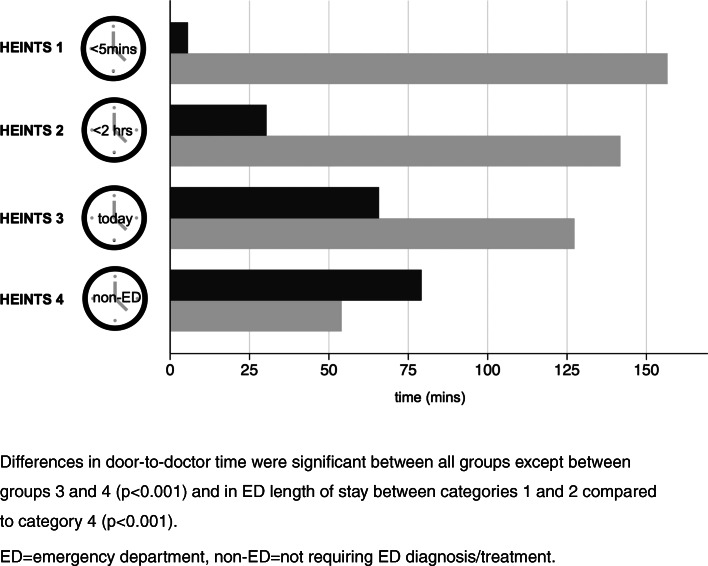
Means of door-to-doctor time (dark grey) and ED length of stay (light grey) in HEINTS triage categories 1 to 4 Differences in door-to-doctor time were significant between all groups except between groups 3 and 4 (*p* < 0.001) and in ED length of stay between categories 1 and 2 compared to category 4 (*p* < 0.001). ED = emergency department, non-ED = not requiring ED diagnosis/treatment

In non-acute patients, there was no significant difference in door-to-doctor time between the groups (pre-HEINTS: 52.7 min ± 8.9, HEINTS: 68.2 min ± 7.2; *p* = 0.177), while LOS was significantly shorter in this subgroup of patients (pre-HEINTS: 270.6 ± 14.8, HEINTS: 114.2 min ± 10.2; *p* < 0.001). In acute patients, however, door-to-doctor time was shorter in HEINTS patients (pre-HEINTS: 41.0 min ± 7.9, HEINTS: 17.7 min ± 2.5; *p* = 0.005) as was LOS (pre-HEINTS: 304.3 min ± 18.3, HEINTS: 149.4 min ± 8.5; *p* < 0.001; Fig. 3). Process times for HEINTS categories are presented in Fig. 4. Significant group differences in door-to-doctor time emerged between all groups except between groups 3 and 4 (d = .47; *p* < 0.001) and in ED LOS between categories 1 and 2 compared to category 4 (d = −.19; *p* < 0.001).

When considering resource use with respect to acuity, fewer laboratory examinations and consultations with other specialties were performed in non-acute patients during the HEINTS phase. Acute HEINTS patients received fewer ECGs in the emergency department than acute pre-HEINTS patients (Table 3).

**Table 3:** Resource utilization in acute and non-acute patients in pre-HEINTS and HEINTS groups

	non-acute			acute		
	pre-HEINTS (*N* = 114)	HEINTS (*N* = 115)	p value	pre-HEINTS (*N* = 181)	HEINTS (*N* = 185)	p value
Resource, *N* (%)						
CT	34 (29.8)	22 (19.1)	0.083	84 (46.4)	70 (37.8)	0.096
MRI	9 (7.9)	8 (7.0)	0.845	58 (32.0)	51 (27.6)	0.348
lumbar puncture	5 (4.4)	3 (2.6)	0.722	10 (5.5)	6 (3.2)	0.316
ECG	64 (56.1)	52 (45.2)	0.144	124 (68.5)	106 (57.3)	**0.031**
laboratory examination	110 (96.5)	99 (86.1)	**0.021**	177 (97.8)	174 (94.1)	0.109
≥1 consultation with another specialty	53 (46.5)	30 (26.1)	**0.002**	83 (45.9)	82 (44.3)	0.194

*CT* computed tomography, *ECG* electrocardiogram, *HEINTS* Heidelberg Neurological Triage System, LWBS left without being seen, *MRI* magnetic resonance imaging

## Discussion

We compared ED process times as well as resource utilization prior to and following the introduction of HEINTS, a dedicated neurological triage system, in an interdisciplinary emergency department.

Acute patients were seen more promptly in the HEINTS phase as an immediate consequence of the HEINTS target times for patient-doctor contact. The HEINTS length of ED stay was shorter for neurological patients both regardless of acuity and when taking acuity into consideration. Length of stay is a central indicator of the quality of ED patient management and flow. It is both cause and consequence of ED crowding [20], which in turn is associated with increasing mortality rates [37]. Data for neurological patients, in particular those with cerebrovascular events, are conflicting in that positive associations between ED LOS and extent of functional outcome [2, 33], no association [7], and even an inverse relation [21] have been observed. While a reduction of ED LOS does not immediately follow from a triage procedure, several factors may contribute to this finding. To begin with, prior to the introduction of HEINTS, the practice of sending patients away after a brief evaluation to have diagnostic work-up and treatment performed in a different setting, e. g. by a practice-based physician, was only rarely and rather unsystematically pursued. Since LOS for category 4 patients was significantly shorter than that of patients of all other triage categories, it can be assumed that the way these patients are handled contributes to an overall reduction of LOS. Acute patients, however, also spent less time in the ED after introducing the triage system. It may well be that the act of triaging according to HEINTS with the ensuing requirements for door-to-doctor time may have increased ED staff’s awareness of the temporal aspect of ED management in general. In addition, the overall reduction of ED resource utilization, which is frequently time-consuming, may have contributed to the reduction of LOS. The time required for advanced imaging procedures as well as the turnaround time of laboratory tests are relevant determinants of ED LOS across different acuity levels [13, 14], so a critical evaluation of their necessity in patients of lower acuity may be not only cost-effective but also contribute to improve ED patient flow. In the same vein, the reduced number of consultations with other specialties – a frequent cause of increased ED LOS [17] – may also have had an impact.

Acuity evaluation of presenting symptoms differed for vertigo, which was more frequently evaluated as acute, and epileptic seizures, which were less frequently evaluated as acute when using HEINTS for triage. It may well be that the different kinds of acuity assessment – retrospective, documentation-based for pre-HEINTS patients versus prospective, HEINTS-based assessment for HEINTS patients – contribute to this finding. Early neurological assessment of a patient presenting with vertigo is useful since it may leave sufficient time for acute therapeutic interventions should the clinical examination and subsequent diagnostic work-up suggest a central cause such as a posterior circulation ischemic stroke or hemorrhage. Established general triage systems like ESI or the MTS assign dizziness or vertigo a lower acuity unless vital signs are alarming. It also has to be noted that subtler abnormalities suggestive of a central pathology, e. g. of the oculomotor system, may be missed in the initial assessment by the triage nurse or non-neurologists [15]. Furthermore, the diagnostic and therapeutic approach frequently taken towards the patient presenting with dizziness is suboptimal [26]. Epileptic seizures, on the other hand, were less often evaluated as acute in the HEINTS phase. An epileptic seizure in a patient with a known history of epilepsy frequently leads to ED admission [8]. With limited information on-scene and the absence of for criteria for non-conveyance, emergency medical service staff often decides to err on the side of safety and transport such a patient to the ED [6, 27] even when their actual condition would not necessitate this. The assignment of a patient with a seizure in the context of known epilepsy to HEINTS category 3 is most likely one reason for this finding of lower acuity in comparison to the pre-HEINTS period. ESI, for example, assigns all patients with a seizure regardless of history and current condition to category 2, demanding physician assessment within ten minutes. Recent studies indicate that most patients with known epilepsy may be managed using outpatient services [8].

There is a large – and in light of recent advances in the treatment of acute ischemic stroke [3, 28] growing – number of stroke-specific tools aimed at the fast and reliable identification of patients with a suspected acute cerebrovascular event both in pre-hospital and ED settings [42]. In addition, various algorithms have been developed for the emergency evaluation of other neurological symptoms, most notably vertigo [10]. An overarching triage algorithm for patients with neurological complaints has been missing up to the development of HEINTS, which is all the more relevant since the representation of neurological symptoms in established general triage systems appears insufficient: on the one hand, a large number of patients fall into one of the more urgent categories with potential over-triage [16], on the other hand, patients are assigned to less acute categories when they present with atypical symptoms or when they are less severely affected [19]. In this regard, a refined approach to the patient with a neurological deficit should be taken not only regarding ED clinical evaluation and diagnostic work-up [23] but even prehospitally and during triage. Our study is still exploratory, and more investigations are required, in particular a performance comparison of HEINTS and instruments like ESI or MTS to determine whether the added complexity, which the use of an additional tool undoubtedly brings, will be outweighted by a more effective and efficient treatment of neurological patients in the ED.

### Limitations

Our results have to be interpreted in the context of several limitations. To begin with, triage in the HEINTS period was done by the ED neurological residents. While this might be deemed a limitation, as most of the triage in EDs is done by nurses, the aspect of triage validity between different personnel was not the focus of our analysis. This not only did not differ from the pre-HEINTS condition but physician-led triage and early assessment has been demonstrated to improve patient outcomes and ED performance [1], both potentially facilitating the assessment of the research questions addressed. Further, acuity assessment of the pre-HEINTS cohort was, of necessity, retrospective, relying on written documentation. Since both analytical and intuitive processes impact on clinical decision-making [29], the retrospective evaluation, lacking the experiential component, will most likely be skewed. In addition, a finer-grained retrospective evaluation of acuity, i. e. more precise than the distinction between “acute” and “non-acute”, which would have been desirable, appears difficult to implement. In order to compare the two retrospective categories with the prospective four-level HEINTS system, levels 1 and 2 as well as levels 3 and 4 were conflated into two acuity levels approximately equating the retrospective categories. We are well aware that, by doing this, very different notions of acuity are subsumed into one category, since a HEINTS category 1 emergency may carry strikingly different diagnostic and/or therapeutic implications than a HEINTS 2 category. The same applies for the fusion of HEINTS categories 3 and 4 into “non-acute” for aforementioned reasons of comparability. We also did not perform investigations to validate HEINTS in our interdisciplinary ED but focused solely on process times and resource use. Finally, differences regarding the structure and organization of emergency departments between different countries also need to be taken into account, limiting generalizability to a certain degree.

## Conclusion

A dedicated triage system for patients reduces DDT, LOS and ED resource utilization. It thereby significantly contributes to improving patient flow and emergency department efficiency. In light of the idiosyncrasies and complexity of many neurological conditions and the challenges arising therefrom, we advocate a refined approach to the patient with a neurological deficit prehospitally and, particularly, during triage.

## Data Availability

The datasets used and/or analyzed during the current study are available from the corresponding author on reasonable request.

## Abbreviations

CT: Computed tomography
CTA: CT angiography
DDT: Door-to-doctor time
ECG: Electrocardiogram
ED: Emergency department
EEG: Electroencephalography
ESI: Emergency Severity Index
HEINTS: Heidelberg Neurological Triage System
IED: Interdisciplinary emergency department
LOS: Length of stay
LP: Lumbar puncture
MRI: Magnetic resonance imaging
MTS: Manchester Triage System
